# Making sense of a new technology in clinical practice: a qualitative study of patient and physician perspectives

**DOI:** 10.1186/s12913-015-1071-1

**Published:** 2015-09-22

**Authors:** Regitze A. S. Pals, Ulla M. Hansen, Clea B. Johansen, Christian S. Hansen, Marit E. Jørgensen, Jesper Fleischer, Ingrid Willaing

**Affiliations:** Health Promotion Research, Steno Diabetes Center A/S, Gentofte, Denmark; Clinical Epidemiology Research, Steno Diabetes Center A/S, Gentofte, Denmark; Medical Research Laboratories, Clinical Institute of Medicine, Aarhus University, Aarhus, Denmark

**Keywords:** Users’ experiences, Technology, Risk assessment, Diabetes, Cardiac autonomic neuropathy

## Abstract

**Background:**

The number of new technologies for risk assessment available in health care is increasing. These technologies are intended to contribute to both improved care practices and improved patient outcomes. To do so however, there is a need to study how new technologies are understood and interpreted by users in clinical practice. The objective of this study was to explore patient and physician perspectives on the usefulness of a new technology to detect Cardiovascular Autonomic Neuropathy (CAN) in a specialist diabetes clinic. The technology is a handheld device that measures resting heart rate and conducts three cardiac autonomic reflex tests to evaluate heart rate variability.

**Methods:**

The study relied on three sources of data: observations of medical consultations where results of the CAN test were reported (*n* = 8); interviews with patients who had received the CAN test (*n* = 19); and interviews with physicians who reported results of the CAN test (*n* = 9). Data were collected at the specialist diabetes clinic between November 2013 and January 2014. Data were analysed using the concept of technological frames which is used to assess how physicians and patients understand and interpret the new technology.

**Results:**

Physicians generally found it difficult to communicate test results to patients in terms that patients could understand and to translate results into meaningful implications for the treatment of patients. Results of the study indicate that patients did not recall having done the CAN test nor recall receiving the results. Furthermore, patients were generally unsure about the purpose of the CAN test and the implications of the results.

**Discussion:**

Involving patients and physicians is essential when a new technology is introduced in clinical practice. This particularly includes the interpretation and communication processes related to its use.

**Conclusions:**

The integration of a new risk assessment technology into clinical practice can be accompanied by several challenges. It is suggested that more information about the CAN test be provided to patients and that a dialogue-based approach be used when communicating test results to patients in order to best support the use of the technology in clinical practice.

## Background

In recent years the number of new healthcare technologies available to the healthcare industry has increased rapidly. Most of these new technologies can be characterized as decision support systems, often utilizing computer applications that are intended to assist health professionals and patients in making decisions about care and intervention options [1, 2]. However, according to Berg et al. [3] a major impediment to the introduction of new technologies in health care practice is the need to integrate new technologies into existing routines [3]. For instance, the technology may seem illogical to the users if the actions prescribed by the technology run against their daily routines [4, 5]. This points to the importance of studying how patients and physicians understand the new technology in relation to their daily practice.

Studies of user perspectives on new healthcare technologies have shown that health professionals and patients may consider new technologies as useful tools assisting health professionals and patients in clinical decision making [2, 6, 7]. However, it has also been shown that health professionals find it difficult to integrate technology into their work practices if there is a mismatch between the expectations of the technology and work practices [8]. This can result in resistance to use the technology, partial use of the technology or other practices to overcome perceived limitations of the technology [8]. In addition, studies have shown that differing priorities between health professionals and patients translate into differing criteria when assessing the usefulness of new technology [2, 6, 7]. Two studies of user attitudes toward a new technology for cardiovascular risk assessment found that the main concern of clinicians was that the use of the system would increase consultation time [2, 6]. In addition, clinicians reported some difficulties using the technology. In a study by Wilson et al. [2], patients found that the program positively impacted the consultation process, allowing them to contribute to the assessment and management of their cardiovascular risk. However, other studies have found that new technologies can interfere with health professional-patient relationships, creating for example, a loss of relational contact [7, 9].

In considering the aforementioned studies it is important to note that new healthcare technologies vary considerably in their design and function [1]. They differ according to the task they are designed to support, the way patient data are utilized, the types of outputs that are generated and the ways outputs are communicated to health professionals and patients [1]. In this study we focus on a new technology designed to detect Cardiac Autonomic Neuropathy (CAN) in people with diabetes.

CAN is a serious and frequently occurring complication of diabetes and has been reported to be a predictor of cardiovascular morbidity and mortality in people with diabetes [10]. It has been shown that CAN is associated with a high risk of cardiac arrhythmias and sudden death, which is possibly related to silent myocardial ischemia [11]. Abnormalities in cardiac autonomic activities can be found in people at diabetes onset and in people with pre-diabetes [12, 13]. Patients are often unaware of having CAN, as the complication may be asymptomatic even long into the course of diabetes [10, 12, 13]. It has been suggested that early detection of CAN is important to motivate patients and physicians to minimize risk factors and thereby reduce further development of complications [14, 15]. It is therefore currently recommended that screening for CAN takes place at point of diagnosis of type 2 diabetes and within five years after diagnosis of type 1 diabetes, to best improve health outcomes [16, 17].

CAN screening can be performed using a recently developed handheld device, the Vagus™ device [14]. The device measures resting heart rate and conducts three different standardized cardiac autonomic reflex tests to evaluate heart rate variability [14]. The tests include measurement of heart rate response during rest, after changing position, during expiration and inspiration and during Valsalva maneuver [14]. The measurement of heart rate variability using cardiac autonomic reflex tests is currently the most sensitive and specific test for CAN [10]. Abnormal heart rate variability in one test indicates early signs of CAN or borderline disease. Two or more tests demonstrating abnormal heart rate variability confirm a diagnosis of CAN [14]. The prevalence of CAN in people with diabetes was recently estimated using the technology and use of the new technology was found to be both feasible in and relevant to clinical practice [14].

The developers of the CAN screening device have described three primary functions of the tool: 1) help refine cardiovascular risk stratification, 2) lead to an increased focus on the prevention of late complications of diabetes, and 3) serve as a tool to engage patients in their own diabetes care^1^.

**Fig. 1:**
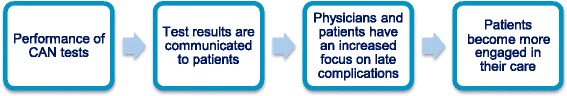
The intended use of the CAN test in clinical practice

The functions described above suggest that the device is intended to support health professionals in clinical decision making and assist patients in their care through detection of CAN Fig. 1,^2^. However, to transform these intentions into practice, the intended use of the technology must be supported by the users and the organizational context of use. This requires that users learn how to utilize the technology and start to actually use it in their daily work [18]. Adoption of a new technology is therefore a continuous process requiring the engagement and empowerment of end-users [1, 19, 20]. To understand whether or not this happens and the process by which it might occur, there is a need to examine how the technology is perceived by physicians and patients in clinical practice and what meaning they ascribe to the technology [21–23]. This includes the processes by which test results are understood and communicated in clinical practice.

The objective of this study was to explore patient and physician perspectives on the use of a new CAN detection technology at a Danish specialist diabetes clinic in the greater Copenhagen area.

### Theoretical framework

The study uses the concept of technological frames to help assess how users understand and interpret the new technology [24]. The concept of technological frames is derived from social cognitive research but also draws on sociological literature exploring the social constructions of technology [24–26]. The notion of technological frames is based on the premise that an individual’s interpretation of new technology is fundamental in influencing how he/she interacts with that technology [24]. An individual’s technological frame is characterized by his or her assumptions, expectations and prior knowledge about a technology, reflecting the process of ‘making sense’ of new technologies [18, 24]. According to Orlikowski and Gash [24] these sense-making processes shape how new technology is used. Furthermore, the authors suggest that different groups within an organization develop different technological frames referring to incongruences between frames. This implies that technological frames are shared by members of a group having a particular interaction with the technology and reflect different ways of knowing and making sense of technology. However, frames can also be inconsistent within a group [24]. The identification of those incongruences and inconsistences between and within user groups can provide an explanation of the difficulties and unanticipated outcomes associated with the introduction of a new technology in an organization. In our study, the concept of technological frames and the identification of incongruences and inconsistencies between and within frames informed the collection as well as the analysis and interpretation of data.

## Methods

The exploration of patient and physician perspectives on CAN test use in clinical practice was carried out between November 2013 and January 2014. The study is based on three sources of data: observations of medical consultations where results of the CAN test were reported (*n* = 8); interviews with patients who had received the CAN test (*n* = 19); and interviews with physicians who reported results of the CAN test (*n* = 9).

### Setting

Data collection was carried oData collection was carried out at a specialist diabetes clinic in the greater Copenhagen area parallel to a quantitativeut at a specialist diabetes clinic in the greater Copenhagen area parallel to a quantitative implementation study of the Vagus™ device at the clinic. This study was initiated as a qualitative contribution to the implementation study to explore user perspectives on the introduction of the device to the clinic within the period of November 2013 and January 2014. The clinic serves as an integrated part of the Danish National Health Service and has a patient base of around 5600 patients with type 1 and type 2 diabetes from the Capital Region of Denmark. Physicians who participated in the study were invited to attend a seminar series over the course of four different mornings at the specialist diabetes clinic. At the seminars, physicians were introduced to CAN and CAN diagnosis. Furthermore, they were shown how to use the device (Vagus™) to conduct a CAN test and were provided with an information sheet outlining the characteristics of patients eligible for the CAN test, diagnostic criteria for CAN and a set of guidelines for using the CAN test in clinical practice. At the clinic, the CAN test was performed by laboratory technicians. Physicians could then access test results through the Electronic Patient Record (EPR) and use the result in medical consultations with patients. Patients eligible for the CAN test received an invitation letter with information on the test.

### Data collection

Patients who received the CAN test and where scheduled to receive the results at a subsequent medical consultation between November 2013 and January 2014 were identified through the EPR (*n* = 55). A patient coordinator at the clinic assisted in identifying those patients. We conducted observations between January 2^nd^ and January 14^th^ 2014. At days where consultations were scheduled, we contacted the respective physicians to request permission to perform observations. In addition, we had correspondence with a nurse who kept us updated about forthcoming consultations where test results were to be provided. A series of observation sessions of medical consultations in which CAN test results were reported (*n* = 8) were organized. However, in one observation it turned out that the patient had not received the CAN test due to complications.

### Sampling

**Fig. 2:**
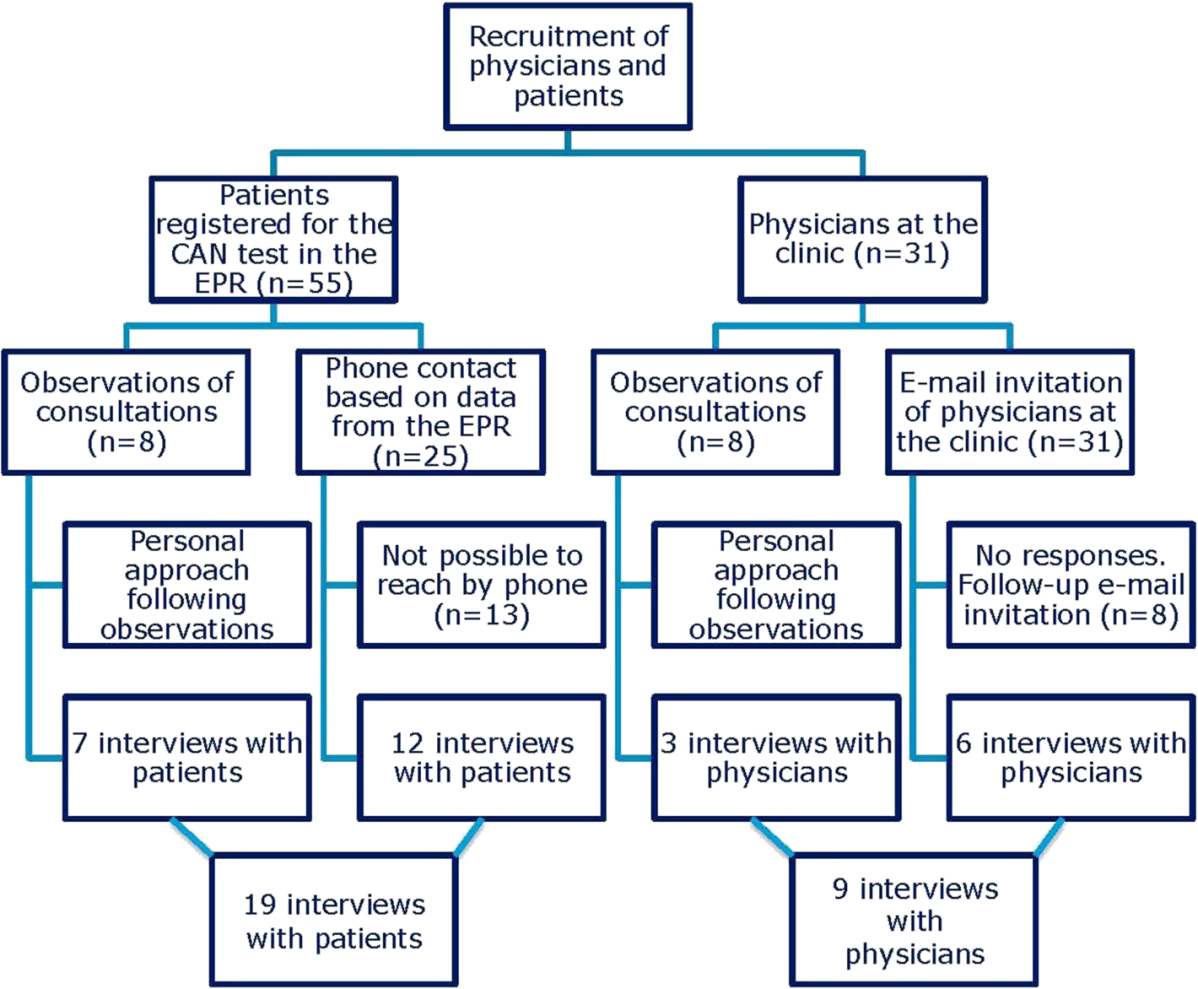
Recruitment of participants for the study

We (RASP, UMH, CBJ) used the following methods to recruit patients and physicians for interviews. Physicians were recruited through 1) personal approach following observations of consultations, 2) e-mail invitations to all physicians at the specialist diabetes clinic. Patients were recruited through 1) personal approach following observations of consultations, 2) phone calls based on data from the EPR Fig. 2,^3^.

After each observation session, the patient and physician were approached with a request to participate in interviews. Using this method, we recruited three physicians and seven patients for interviews.

In parallel with observations, we invited all physicians at the specialist diabetes clinic (*n* = 31) by e-mail to participate in interviews. None of the invited physicians responded to the request and in a purposeful sampling process we recruited eight physicians for a further follow-up invitation. Selection was based on physicians’ level of clinical experience ensuring that both chief physicians and less experienced physicians were invited. In addition, we selected physicians assumed to have more consultations with patients and thus have more frequent communication of test results. Of these eight physicians, six agreed to participate. Combined with the three physicians recruited from observation sessions, interviews were conducted with nine physicians in total. To recruit patients for interviews besides the seven patients recruited at observations, we retrieved phone numbers of patients identified through the EPR. We contacted all patients identified through the EPR whose phone numbers we were able to retrieve (*n* = 25) and conducted an additional 12 phone interviews between January 14^th^ and January 31^st^ 2014. The 13 remaining patients could not be reached by phone. None of the patients refused to participate.

### Observation and interview guides

The primary focus of the observations was to study physicians’ use of the results of the CAN test in clinical practice and to study the communication of test results. At each observation session the observer (RASP, UMH, CBJ) collected field notes based on an observation guide. The observation guide addressed the amount of time spent on communication of test results, the explanations provided by physicians, the dialogue and terminologies used in the communication of test results as well as patients’ reaction to the information provided.

We (RASP, UMH, CBJ) used semi-structured interview guides for interviews with physicians and patients. The interview guides were based on the concept of technological frames focusing on assumptions, perceptions and knowledge of the introduction of the CAN test. This included implications for clinical practice and the communication of test results. Physicians, patients and the interviewer were allowed to develop or introduce new themes. The duration of each interview was about 30 min (mean: 22 min). Interviews were audio-taped and transcribed.

Informed consent was obtained from physicians and patients prior to observations of medical consultations and conducting interviews. Written consent was obtained for interviews in person and verbal consent for phone interviews. The study was accepted by the National Committee on Health Research Ethics and the Danish Data Protection Agency.

### Analytical approach

Patient and physician perspectives on the CAN test were analysed qualitatively. All data including interviews and field observations were analysed using content analysis to systematically make inferences about the intentions and interpretations of physicians and patients as described by Agar [27] and Eisenhardt [28]. In the procedure, we focused on identifying statements or actions reflecting physicians’ and patients’ technological frames. This includes perceptions and knowledge of the CAN test in terms of its implications for clinical practice and the communication of test results. The examination of data was carried out in the following steps in accordance with Orlikowski and Gash [24]: 1) separating the data into statements and actions of physicians and patients respectively, 2) sorting the data into categories suggested by the data, 3) comparing categories generated by the data of physicians and patients to identify common themes, 4) recoding of data using the proposed themes. Using this iterative approach we aimed to determine a set of themes that constituted core domains of physicians’ and patients’ technological frames. During the analysis process we reflected on our pre-assumptions as well as whether our presence affected the clinical performance.

## Results

A total of 19 patients were interviewed. Age of patients ranged from 36 to 79 years (mean age: 64). Three patients were female, and 16 were male. 16 were diagnosed with type 2 diabetes and three with type 1 diabetes. The duration of disease among patients with type 2 diabetes ranged from two to 35 years (mean duration of disease: 12 years). Among patients with type 1 diabetes, the range of disease duration was four to 43 years (average disease duration: 24 years). A total of nine physicians were interviewed. Six physicians were female, and three were male. Three were chief physicians and six were less experienced physicians. Characteristics of the study population are shown in Table 1.

**Table 1:** Characteristics of the study participants

Patients with diabetes	Number
Female/male	3/16
Age (mean)	36-79 years old (64 years old)
Diabetes type 1/2	3/16
Years with diabetes (mean)	
• Type 1	• 4–43 years (24 years)
• Type 2	• 2–35 years (12 years)
Physicians	N
Female/male	6/3
Chief physicians/less experienced physicians	3/6

Of the 19 patients interviewed, four patients did not go through the CAN test due to e.g. physical disability, two patients had not received their test result, one patient did not have the test result registered and eight patients did not remember whether they had received a test result leaving four patients who could actually remember and relate to the CAN test.

Our findings emphasize that the usefulness of a new technology in clinical practice depends on users’ perceptions on how it can be translated into their daily practice. In our analysis, three central concepts emerged: 1) perceptions of the clinical relevance of the CAN test and 2) communication of complex test results and 3) motivation for behavioural change. The three central concepts are closely interrelated in the sense that physicians’ perceptions on the clinical relevance of the test affect the communication of test results. Furthermore, the interaction between patients and physicians can influence whether patients perceive test results as a means to change behaviour. However, we find it useful to analytically distinguish between concepts to highlight relevant differences between patients’ and physicians’ interpretations of the CAN test.

### Perceptions on the clinical relevance of the CAN test

Physicians perceived the CAN test as appropriate for cardiovascular risk stratification, as illustrated in the following excerpt:*“…the good thing about it [CAN test] is exactly that it screens them [patients without symptoms] rather than we just consider them in cases where we think of symptoms that the patient might have…or where we are skilled enough to ask about it every time. It is not everyone with gastroparesis or something alike who notice it until it has gone very far”* (physician 1). Additionally, physicians perceived it as a replacement for existing tests to diagnose CAN in patients: *“You could say that it [the CAN test] is a smart way of having an overall package rather than having [patients] to go through several different examinations”* (physician 2).

However, some physicians suggested that the CAN test is only a clinically relevant tool if it is used to confirm CAN in patients already suspected of having the condition – not a test to be performed indiscriminately as a broad screening for CAN in people with diabetes. As one physician noted:*”It is clinically relevant if there are clinical signs of it [CAN], then it [CAN test] is some sort of confirmation. But to use it for everybody? My personal opinion is that it should be used when relevant”* (physician 3).

Most patients did not recall being tested for CAN or receiving test results from their physician. They suggested that it could be difficult to find information about the test and to distinguish the CAN test from other tests at the specialist diabetes clinic. Patients also felt that too much information was provided at once when visiting the specialist diabetes clinic and that the interval between testing and receiving results was sometimes as long as five months, which may have increased their “forgetfulness”.

Some patients remembered part of the process of taking the test but did not recall or know how the device worked or the reason for being tested. One patient reported the following misunderstanding*:”I did not even know that they tested my internal nervous system. That was not mentioned. I got the impression that it had to do with testing my lung function”* (patient 1). Patients who could recall the test indicated that they had difficulty understanding the purpose of the test, as illustrated in the following excerpt: *“It makes sense if you are told ‘you have this and this condition and you can do this and that’. It is no good that the doctor goes ‘you have this condition and there is nothing you can do about it’. It does not make any sense”* (patient 2).

### Communication of complex test results

Although physicians considered the test useful, some physicians expressed communicating results from the CAN test to patients to be a complicated task. They found it difficult to explain the nature of CAN and especially the implications of a pathological or borderline test result. One physician explained: *“It is a test that is difficult to report because it simply can be difficult to explain the patients about autonomic neuropathy. I spend relatively long time talking about it”* (physician 4).

Other physicians did not report experiencing difficulty communicating CAN test results to patients. However, they reported needing more time to communicate a pathological test result than a non-pathological test result because the former would generate questions about potential implications. One physician noted: *“It is time consuming and it is not the only time consuming procedure here. So you might sometimes wonder how much more they think can be squeezed into fifteen minutes”* (physician 5). Observations generally revealed that physicians assessed and compared the value of using consultation time to explain CAN test results with that of other pressing issues.

Our observations showed that physicians communicated test results in different ways. The process varied according to physicians’ perceptions of patients’ need for information, which affected the way test results were explained and the amount of information provided on the physiological basis of the test. As one physician noted: *“The patients don’t care about stuff like heart rhythm variability I guess. And maybe they don’t care about the long explication and just want to know that it looks fine. But I have this idea of inviting them to a deeper talk about it to understand what we are talking about”* (physician 6).

Other physicians were particularly sensitive to terminology choice when discussing results with patients as a means of facilitating the highest level of understanding in the patient: *“I try to listen to the patient and see if he or she understands what I say. Otherwise I must say something that suits the patient. I try to use words that are closely related to the professional terms that can be understood”* (physician 7). Nonetheless, some patients still suggested that it could be challenging for physicians to translate their professional understanding of test results into terms that would be meaningful and comprehensible to patients: *“Things are self-evident and clear when you are an expert in a field, and so it can be difficult to explain the patient why you measure something specific and what that means (…) the physician has to be very pedagogical”* (patient 3)*.* Our observations showed that most physicians did not systematically investigate whether patients understood their message, which indicated a communication process characterized by a lack of dialogue.

Some physicians did not want to create undue concern in patients by communicating test results: *“If a patient has a pathological test, then it might worry the patient’will I collapse? Will I die?’ Because it is not something we can really fix. You need to communicate in a way so you don’t unduly cause concern but makes the test result usable in a positive sense”* (physician 2)*.*

Overall, observations indicate that physicians found it challenging to communicate test results to patients. Additionally, they were cautious about discussing test results if they felt they could not translate them into meaningful and clearly defined course of action for patient treatment. These results suggest that the technological frames of physicians contrast the technology developer’s intentions in using the tool for risk assessment and encouraging preventive behaviours in patients. The technological frame of physicians was clearly reflected in patients’ knowledge and interpretations of the CAN test.

### Motivation for behaviour change

As illustrated in the following excerpt from an interview, a common challenge articulated by physicians was the difficulty experienced in translating CAN test results into meaningful implications for patient treatment: *“If you inform people about such thing as autonomic neuropathy, then they will always like to know if there is something we can do about it. And that is difficult in my view”* (physician 5)*.* Some physicians however, believed that results from the CAN test would motivate patients to better manage their diabetes or would serve as an incentive for taking medications or promote behaviour change. As one physician explained: *“If you have a patient with an increased cardiovascular risk, then there is a clear communicative effect of telling [the patient] that we have performed this test that clearly indicates that you need blood pressure-lowering and cholesterol-lowering [medicine]. I think that the patient will pay close attention then”* (physician 1). Another physician suggested that the test could encourage decision-making surrounding treatment, explaining: *“It clearly motivates the patient and I to address additional lifestyle changes. You [the physician] can use the test result in relation to your own practice. You are often in a situation where you doubt whether you should initiate a new treatment or not (…) it is clear that it may encourage a decision-making process”* (physician 6).

These physicians attributed a clinical value to the test in terms of using it to justify decisions about care and in this case to serve as an incentive for changing behaviour and medication plans of patients. However, none of the patients indicated that the test results would motivate them to change behaviour. A patient who received a borderline test result reported the following: *“The result will not affect how I handle my diabetes at all; because it was borderline; and there is always a statistical uncertainty (…) I cannot respond to it when it is a threshold value”* (patient 3).

The potential of the CAN test to induce behaviour change was also challenged by the finding that many patients experienced difficulty remembering whether or not they had taken the test and in understanding the implications of test results. Additionally, the meaning that patients ascribed to the test was strongly influenced by their communication with physicians about test results and thus by physicians’ perceptions of the test. Patients found it frustrating to receive a pathological or a borderline test result if they were left with no clear instructions about how to act on CAN diagnosis or with a diffuse risk of something. One patient noted the following: *“It was odd that no more time was spent on explaining what it [CAN] was and what you occasionally could use the results for”* (patient 4). Patients’ and physicians’ attitudes toward the CAN test could therefore be a barrier to using the CAN test as a form of motivation for self-management as intended by the developers of the technology. Most prominently, patients requested more information about the test and the implications of it.

## Discussion

The results of this study reveal that the majority of participating physicians perceived the CAN test as an appropriate tool for cardiovascular risk stratification. Additionally, physicians perceived the test as helpful in supporting decisions about care. However, some physicians found the test to be relevant only in instances where the patient already exhibits symptoms of CAN. Furthermore, it was challenging for physicians to communicate test results in terms that were meaningful and comprehensible to patients. Most patients did not recall being tested for CAN or receiving the test results from their physician. Those who did recall receiving the test were unsure about the purpose of the test and the implications of the results. These results reflect that physicians and patients had different technological frames with regard to their understanding of the vision behind the introduction of the CAN technology (the clinical relevance of the CAN test) and their understanding of how to apply the technology into their daily practice (the communication and interpretation of test results). In addition, we identified inconsistencies with regard to the technological frame of physicians as physicians had different perceptions on the clinical relevance of the CAN test. If these differences between and within technological frames are not articulated, they may hamper the implementation of the technology as was the case in this study.

In accordance with our findings, other studies have found that the use of a technology in clinical practice can be challenged by the perceptions of health professionals [2, 6, 29]. Short et al. [29] studied perspectives of general practitioners on the adoption of a computerized risk assessment system in general practice and identified several perception-based barriers to the use of the system. In line with our findings, general practitioners found it difficult to communicate the output of the risk assessment to patients. Furthermore, the general practitioners expressed concern about explaining risk to patients and reported that the system required extra time in consultations.

In other studies, technologies for risk assessment have received more positive feedback from patients and general practitioners, which proved to enhance the acceptance and use of those technologies in clinical practice [2, 6]. For instance, Carroll et al. [6] involved clinicians and patients in designing and evaluating a decision support system that deals with cardiovascular risk prevention in people with type 2 diabetes [6]. Clinicians and patients were enthusiastic about the system and felt confident using it after short training periods. However, in accordance with the findings of our study, some patients had difficulties in interpreting the clinical results [6].

Gillespie [30] studied how risk was experienced by people who were designated as being at risk of either coronary heart disease or prostate cancer. One of his main findings was that uncertainty was a fundamental aspect of the experience of risk. Respondents were not sure how to react to being at risk, were unsure of the implications being at risk had for their everyday lives and were not given clear instructions on how to address their risk status [30]. These findings have been corroborated in other studies [31, 32]. Gillespie [30] argues that the uncertainty led to patient fear, anxiety and uneasiness about the future and that respondents were unable to rely on self-monitoring of health through physical symptoms. These observations support our finding that the perceived uncertainty about the implications of the CAN test could be a barrier to using the CAN test as a motivation for self-management. However, given the significant lack of recall in patients receiving the test result, the CAN test did not seem to produce fear or anxiety, but rather a request for more information on the test and the meaning of results.

Our findings also indicate that physicians’ and patients’ attitudes varied widely with respect to the perceived importance and implications of the CAN test. This both concerned perceptions within the group of physicians and between patients and physicians. Other studies have identified different perceptions among physicians of the use and value of technologies for risk assessment in health care practice [2, 33]. For instance, some physicians preferred to use their own questions rather than questionnaires [2]. In addition, wide variations in physicians’ interpretation of risk have been demonstrated [33]. This can in turn affect how physicians communicate risk to patients and patients’ understanding of risk, which is also reflected in our study.

Discrepancies between patients’ and physicians’ perceptions of clinical phenomena have also been documented elsewhere [34–36]. It has been shown that health professionals value the quality of their own information more positively than patients and that health professionals to a higher degree than patients experience that specific information was delivered to patients [35] Additionally, a study on patients’ and health professionals’ ability to recall information provided in consultations revealed that patients were unable to call to mind even half of the topics discussed and could recall even fewer decisions that were made about their treatment than did health professionals [36]. According to the authors, this lack of agreement could be related to health professionals bringing their own agendas into the consultation. However, it is also likely that the “forgetfulness” of patients in our study was influenced by other factors. Possible factors include organizational issues in terms of patients being overloaded with information during their visits at the specialist diabetes clinic as well as the knowledge, personal experiences, age and educational level of patients [37].

### Practice implications

The findings of our study point to the importance of acknowledging user perceptions of a technology before and during the process of implementation. The identification and discussion of technological frames of different user groups may reduce the likelihood of misunderstandings and difficulties around the use of new technologies. With regard to this study, it is likely that the identified barriers could have been articulated and discussed at an earlier point in time if patients and physicians were consulted before the CAN technology was introduced to the specialist diabetes clinic. This could imply the development of guidelines around the communication about the CAN test in clinical practice.

In response to the barriers that physicians and patients perceive in relation to the use of the CAN test in clinical practice, the findings of this study suggest that communication and information about the CAN test could be improved. Improvements could be achieved through the development of guidelines about a dialogue-based approach to the communication of test results which could prove useful on how to provide clear and consistent information about the test. The communication between patients and physicians about the CAN test could thus be enhanced through dialogue targeting patient experiences of the test and prompting patients to express their thoughts and expectations. This includes the development of health professionals’ communication skills e.g. skills to include patients in clinical decision making and encourage patient autonomy [36]. It has been shown that patient autonomy is associated with increased motivation, better self-care and metabolic control in patients [38]. This suggests a need to develop physicians’ skills to encourage patient autonomy with regard to the CAN test. In this study, we did not look into specific behaviours or skills that could serve this purpose, but a number of related issues have been discussed in the literature. This includes skills to facilitate a person-centred approach to communication e.g. asking patients about their concerns, expectations and perceptions of the problem in relation to their functioning.

An example of such an approach to the use of a technology for cardiovascular risk assessment is described by Wilson et al. [2]. The technology builds on sharing management and partnership between patients and physicians as well as the promotion of healthy lifestyles. Key features include goal setting, written management plans and regular follow-up. These features could inform use of the CAN test in clinical practice at the specialist diabetes clinic. For instance, physicians can support patients with a pathological or borderline test result by collaborating with them to develop an action plan, which can allow them to feel a sense of control over their disease. In case of a non-pathological test result physicians could invite patients to enter into discussion about their current diabetes-related behaviour and how to maintain or improve it. Physicians must tailor the amount of information they provide to the patient’s response. In addition, a vocabulary of common terminologies applied to the invitation letter for the test, the testing process, and communicating results is suggested so that clear and consistent information is available to patients.

On an organizational level, assessing available resources and competencies and existing priorities of the intended users of the technology can promote and improve the use of the technology in clinical practice. In this case that includes the provision of training for improving communication about the CAN test in medical consultations considering the short time frame of medical consultations. Thus, the potential of the suggestions for improving the translation of the technology into clinical practice can only be realized in collaboration with the users in the specific context of use.

### Limitations

Inclusion of more individuals who tested positive for CAN could have enhanced our insights about this group of patients. However, there were surprisingly many patients who did not recall the test and therefore were not able to elaborate on their experiences with the CAN test. It is also likely that the physicians included in the study represent a selected group due to the recruitment process where only few of the invited physicians participated. With regard to the analysis, the three central concepts identified are much intertwined and thus difficult to distinguish from one another in terms of defining what constitutes the technological frames of patients and physicians respectively. Furthermore, we did not compare the technology with other well-known technologies used for a longer period of time in the clinic.

## Conclusions

This study emphasizes the importance of investigating patients’ and physicians’ understanding of a new technology when it is implemented in clinical practice. The findings reveal that physicians found it challenging to communicate the meaning of test results to patients and to translate results into meaningful implications for patient treatment. Patients were generally unsure about the meaning of test results and did not indicate that test results would motivate them to change behaviour. In order to support the use of the technology in clinical practice, it is suggested that a dialogue-based approach be used when communicating test results to patients including gaining impression of the patient’s understanding of the purpose of the CAN technology. In addition, it is suggested that more information about the CAN test should be provided to patients.

## Abbreviations

CAN: Cardiac Autonomic Neuropathy

## Footnotes

1 These visions are described in a presentation by the person responsible for implementing the technology at the specialist diabetes clinic and were presented at settings within and external to the clinic.

2 Fig. 1 illustrates the anticipated use of the technology at the specialist diabetes clinic.

3 Fig. 2 illustrates the recruitment of participants for the study.
